# Relationship between the extent of aortic replacement and stent graft for acute DeBakey type I aortic dissection and outcomes: Results from a medical center in Taiwan

**DOI:** 10.1371/journal.pone.0210022

**Published:** 2019-01-04

**Authors:** Chiao-Po Hsu, Chun-Yang Huang, Fei-Yi Wu

**Affiliations:** 1 Department of Medicine, School of Medicine, National Yang-Ming University, Taipei, Taiwan; 2 Division of Cardiovascular Surgery, Department of Surgery, Taipei Veterans General Hospital, Taipei, Taiwan; 3 Department of Surgery, Taoyuan General Hospital, Ministry of Health and Welfare, Taoyuan, Taiwan; Case Western Reserve University School of Medicine, UNITED STATES

## Abstract

**Background:**

Total arch replacement (TAR) and/or stent graft implantation has been proposed as the primary surgical treatment for acute DeBakey type I aortic dissection. However, the suggestion was based on excellent outcomes of high-volume or aortic centers. How about the real results in most places around the world? The purpose of this study was intended to compared in-hospital mortality, major complications, and aortic remodeling between TAR and/or stent graft implantation in a medical center of northern Taiwan.

**Methods:**

Between January 2008 and August 2017, 156 patients with acute type I aortic dissection underwent surgery at our institution, including proximal aortic replacement only (Group I, n = 72), concomitant TAR (Group II, n = 23), concomitant TAR extended with stent grafting (Group III, n = 45), and proximal aortic replacement with descending aortic stent grafting (Group IV, n = 16).

**Results:**

No significant differences were found in underlying disease and preoperative presentations, including operative risk among four groups. Overall in-hospital mortality was 22.4% (13 patients in Group I, 9 in Group II, 12 in Group III, and 1 in Group IV). New-onset stroke occurred in 15 patients postoperatively (3 patients [5.2%] in Group I, 3 [21.4%] in Group II, and 9 [26.5%] in Group III after excluding 36 patients with documented preoperative cerebrovascular accident or cerebral malperfusion). Root reconstruction and TAR were significantly associated with in-hospital mortality. TAR was significantly associated with surgery-related stroke. Compared to those in Group I, true lumen expansion and false lumen shrinkage during 1-year aortic remodeling were significantly higher in Groups III and IV. Both TAR and descending aorta stent grafting were significantly associated with decreased risk of patent false lumen.

**Conclusions:**

Proximal aortic replacement remains the preferred surgical strategy for acute type I aortic dissection, with lower mortality and neurological complications. Proximal descending aorta stent grafting may benefit aortic remodeling, even without TAR.

## Introduction

Acute DeBakey type I aortic dissection remains a surgical challenge for cardiothoracic surgeons, and determining the extent of aortic intervention is a critical step. Proximal aortic replacement (PAR; that is, replacing the ascending aorta with or without extension to the hemiarch) may cause late sequelae, including persistent distal patent false lumen (PFL), aneurysmal enlargement, and possible repeat surgery [1]. The incidence of such sequelae is reduced using concomitant total aortic arch replacement (TAR) [1]. Because concomitant TAR has a significantly higher operative mortality [2, 3], type I aortic dissection is usually treated using PAR only unless the primary tear is in the arch.

Over the last 2 decades, developments in stent grafting techniques have improved the management of aortic diseases, reducing mortality and morbidity of conventional aortic surgical repair [4, 5]. Some groups have advocated a more aggressive approach in which routine TAR and/or extending with a stent graft as the primary surgical strategy for type I aortic dissection [6–8], as supported by recent meta-analyses [9, 10]. However, these meta-analyses reviewed data from high-volume centers and non-randomized data sets, and the excellent outcomes of TAR and stent grafting may not be applicable to most patient populations across the world. These studies might lead surgeons to risk patient safety in expectations of remarkable patient benefits.

How about the real outcomes of operation for type I aortic dissection in most places around the world? In clinical practice, no one method can be always applied, given the variety of clinical presentations and several factors, such as age, primary tear location, arch diameter, malperfusion syndrome, and technical improvements such as the stent graft that was widely used in the past decade. The stent graft was released in 2008 in Taiwan for use in aortic pathology, including aortic dissection. This precludes sample randomization, and hence retrospective analysis would be more appropriate. The purpose of this retrospective study was to assess the effect of extended aortic replacement and/or stent grafting on the outcomes of patients with acute type I aortic dissection in the past decade. We compared in-hospital mortality, major complications, and remodeling of the proximal descending aorta.

## Methods

### Patients

This retrospective study was approved by the Institutional Review Board at Taipei Veterans General Hospital (approval number: 2015-11-009BC) and informed written consent to access their medical records was obtained from each patient. Between January 2008 and August 2017, 156 patients with acute type I aortic dissection were admitted to Taipei Veterans General Hospital, a medical center in Northern Taiwan. Diagnosis was confirmed in all patients using computed tomography (CT) scans before performing emergent surgery (less than 2 hours after arrival to the emergency department). Surgical procedures and extent of aortic replacement were determined by the on-duty surgeon.

Patients were categorized into 4 surgical groups according to TAR and/or implantation of stent graft in the descending aorta: Group I, proximal aortic replacement (PAR) only (ascending aorta or extension to hemiarch); Group II, concomitant TAR; Group III, extended TAR with concomitant stent grafting; Group IV, PAR and descending aorta stent grafting.

### Surgical procedures

All patients underwent median sternotomy. Cardiopulmonary bypass involved cannulation of one side of the femoral artery and/or the right axillary artery. The right axillary artery was used for antegrade cerebral perfusion, and the superior vena cava was used for retrograde cerebral perfusion. Cerebral perfusion was monitored using intraoperative cerebral oximetry (INVOS Cerebral/Somatic Oximetry, Covidien-Medtronic, Minneapolis, MN, USA). For stent graft implantation, a soft guidewire was cannulated retrogradely through the femoral artery to the true lumen of the aortic arch under fluoroscopy guidance, with confirmation using transesophageal echocardiography.

### Proximal anastomosis

Proximal anastomosis was usually performed first. The aorta was cross-clamped and the cardioplegia solution was perfused antegradely directly through the coronary ostia. A Dacron graft was anastomosed proximally at the sinotubular junction of the ascending aorta. If necessary, aortic valve treatment was performed by valve resuspension, root reconstruction, or valve replacement during cooling phase.

Once an adequate core temperature (22°C-28°C) was achieved, circulatory arrest was initiated and the aortic clamp was removed, followed by retrograde or antegrade cerebral perfusion.

### Group I

A straight Dacron graft was used for proximal aortic replacement. After completing open distal anastomosis, air was removed through a venting cannula placed on the anterior aspect of the graft. Subsequently, systemic perfusion was resumed and the surgery was completed in a routine manner.

### Group II

During circulatory arrest with antegrade cerebral perfusion, aortic transection was usually performed beyond the origin of the left subclavian artery (LSA). An oval opening was made where the supra-aortic arteries were re-implanted as one island (island technique) or each supra-aortic artery was cut for re-implantation individually (branched graft technique). A straight graft (or multiple branches) of appropriate length was first anastomosed to supra-aortic arteries [11], then antegrade cerebral perfusion was also performed through the graft. Another straight graft of appropriate length was anastomosed to the proximal descending aorta. The elephant trunk technique was often used for this anastomosis. A straight graft of appropriate length (rolled inside-out as a sleeve graft) was inserted into the aorta. The distal end was running-sutured and reinforced with Teflon felt outside the aortic wall. The free end of the reversed sleeve graft was then withdrawn. The graft connected to the proximal thoracic aorta was end-to-end anastomosed to the graft connected to the aortic root, followed by end-to-side anastomosis with the graft connected to supra-aortic arteries.

### Group III

The procedure in Group III was mostly similar to that in Group II. The difference, however, was that during distal anastomosis, the soft guidewire was pulled out, held tightly, and the 15-cm stent graft (TAG, Gore Inc, USA) was deployed antegradely in the elephant trunk through the guidewire under direct vision.

### Group IV

Similar to the procedure mentioned in earlier reports [6, 12], during circulatory arrest with antegrade cerebral perfusion, the 15-cm stent graft (TAG, Gore Inc, USA) was deployed antegradely through the guidewire into the proximal thoracic aorta distal to the orifice of the LSA under direct vision. The diameter of the stent graft was determined by adding around 5%-10% to the outermost diameter of the proximal descending aorta. The subsequent procedure was similar to that in Group I.

### Follow-up

Surviving patients received regular follow-up in the outpatient department, including postoperative surveillance with contrast CT performed initially within 1 month and at least once every year thereafter.

### Surgery-related stroke

To evaluate surgery-related stroke, patients with a history of preoperative cerebrovascular accident (CVA) and newly developed neurologic deficits because of cerebral malperfusion were excluded. Postoperative new-onset stroke was considered as surgery-related stroke and confirmed based on clinical findings and diagnostic imaging.

### Aortic remodeling

Digital Imaging and Communications in Medicine data were transferred to OsiriX MD (OsiriX Version 1.1, Pixmeo, Switzerland) for evaluation, and volumes were computed automatically using the region of interest. False lumen status in the descending aorta on CT images was classified as “no” or “total” thrombosis as applied to stent graft coverage or comparative descending aorta post operatively. During volumetric analysis, both true and false lumen volumes of the descending aortic segment from the subclavian artery to the celiac trunk were measured and compared with those on the preoperative CT scan at the same level. The volumetric change ratio was calculated as (Volume_1year_/Volume_preop_) − 1

### Statistical analysis

All continuous variables are presented as mean ± standard deviation and categorical variables as numbers and percentages. For continuous variables, the Kolmogorov-Smirnov method was used for initial normal distribution analysis. The Mann-Whitney U test or T test was used to analyze differences between survivors and non-survivors. For categorical variables, the chi-square test was used. One-way analysis of variance was applied for evaluating aortic remodeling. Multivariate logistic regression analysis was performed to determine independent risk factors of outcomes after backward stepwise-selection method. Odds ratios (ORs) and 95% confidence intervals (CIs) were calculated. A p-value <0.05 was considered statistically significant. Data were analyzed using SPSS statistical software (version 22.0; IBM Corp, Armonk, NY, USA.)

## Results

A total of 156 patients received emergent surgery for acute type I aortic dissection: 72 patients in Group I, 23 in Group II, 45 in Group III, and 16 in Group IV.

The clinical and surgical characteristics of the 4 groups were compared and are summarized in Table 1.

**Table 1:** Clinical and operative characteristics of four surgical groups.

Clinical Characteristic	I(N = 72)	II(N = 23)	III(N = 45)	IV(N = 16)	P
Age (years)	57.9±12.6	61.2±11.8	57.2±12.8	58.8±12.4	0.643
Gender(M/F)	50/22	15/8	31/14	10/6	0.942
Hypertension	60(83.3%)	20(87.0%)	32(71.1%)	11(68.8%)	0.229
Smoking	20(27.8%)	6(26.1%)	17(37.8%)	4(25.0%)	0.611
Coronary artery disease	11(15.3%)	0(0)	7(15.6%)	2(12.5%)	0.073
Hyperlipidemia	3(4.2%)	1(4.3%)	3(6.7%)	1(6.3%)	0.937
Chronic obstructive pulmonary disease	5(6.9%)	3(13.0%)	1(2.2%)	1(6.3%)	0.378
Diabetes mellitus	8(11.1%)	3(13.0%)	6(13.3%)	1(6.3%)	0.870
Cerebrovascular accident	6(8.3%)	4(17.4%)	7(15.6%)	0(0)	0.112
Previous cardiac surgery	0(0)	0(0)	1(2.2%)	0(0)	0.475
Previous aortic surgery	2(2.8%)	0(0)	2(4.4%)	0(0)	0.464
Chronic kidney disease	2(2.8%)	1(4.3%)	4(8.9%)	0(0)	0.304
Hemodialysis	0(0)	0(0)	2(4.4%)	0(0)	0.169
Marfan syndrome	4(5.6%)	0(0)	2(4.4%)	0(0)	0.308
Malperfusion	27(37.5%)	12(52.2%)	22(48.9%)	3(18.8%)	0.112
IA dissection	25 (34.7%)	10 (43.5%)	24 (53.3%)	5 (31.3%)	0.196
LCCA dissection	22 (30.6%)	14 (60.9%)	15 (33.3%)	6 (37.5%)	0.066
LSA dissection	20 (27.8%)	12 (52.2%)	13 (28.9%)	6 (37.5%)	0.156
Operative Characteristic					
Brain protection (antegrade/retrograde)	49/23	22/1*	45/0*	16/0*	<0.001
Cardiopulmonary bypass(min)	244.3±52.3	336.0±91.1*	317.6±92.8*	273.5±58.7^#^	<0.001
Aortic clamp(min)	124.8±32.7	201.8±52.4*^#^	166.9±49.3*	147.5±46.0	<0.001
Circulatory arrest(min)	36.0±12.5	71.4±22.9*^#^	53.9±31.4*	53.6±29.0*	<0.001
Primary tear location					0.195
Ascending aorta	41 (56.9%)	15 (65.2%)	25 (55.6%)	9 (56.3%)	
Arch (lesser curvature)	9 (12.5%)	2 (8.7%)	5 (11.1%)	2 (12.5%)	
Arch (great curvature)	4 (5.6%)	4 (17.4%)	9 (20.0%)	3 (18.8%)	
Proximal descending aorta	6 (8.3%)	1 (4.3%)	4 (8.9%)	2 (12.5%)	
unknown	12 (16.7%)	1 (4.3%)	2 (4.4%)	0 (0)	
Primary tear resection	48 (66.7%)	22 (95.7%)*	37 (82.2%)	11 (68.8%)	0.022
Root reconstruction	15(20.8%)	7(30.4%)^#^	3(6.7%)*	3(18.8%)	0.061
CABG	1(1.3%)	1(4.3%)	0(0)	0(0)	0.451
ASA classification (III/IV/V)	3/60/9	1/20/2	1/36/8	1/14/1	0.867

ASA: American Society of Anesthesiologists; CABG, Coronary artery bypass graft; IA, innominate artery; LCCA, left common carotid artery; LSA, left subclavian artery; Age, body temperature, cardiopulmonary bypass, aortic clamp, circulatory arrest by T test (normal distribution analysis by Kolmogorov-Smirnov test and Post Hoc test) and other variables by Chi-square test.

* p <0.05 vs. Group I

^#^ p <0.05 vs. Group III.

No significant differences were found in underlying disease and preoperative presentations, including operative risk predicted by ASA (American Society of Anesthesiologists) classifications, among the 4 groups; however, significant differences were seen in several intraoperative variables. Retrograde brain protection was used for almost all patients in Group I. The durations of cardiopulmonary bypass, aortic clamping, and circulatory arrest were longer in Groups II and III than those in Group I. There was no difference in concomitant surgery, including coronary artery bypass graft and aortic root reconstruction.

Table 2 provides a comparison of postoperative mortality and morbidity in the 4 groups.

**Table 2:** Postoperative mortality and 30-day morbidity of four groups.

Operative characteristic	Group I(N = 72)	Group II(N = 23)	Group III(N = 45)	Group IV(N = 16)	P
Mortality	13(18.1%)	9(39.1%)	12(26.7%)	1(6.3%)	0.062
Group I		0.037*	0.269*	0.450*	
Group II			0.293*	0.028*	
Group III				0.153*	
Stroke	10(13.9%)	8(34.8%)	16(35.6%)	1(6.3%)	0.008
Group I		0.035*	0.006*	0.681*	
Group II			0.950*	0.056*	
Group III				0.027*	
Acute kidney injury	16(22.2%)	7(30.4%)	13(28.9%)	4(25.0%)	0.808
Ischemic bowel	2(2.8%)	0(0)	2(4.4%)	0(0)	0.464
Re-exploration for bleeding	7(9.7%)	2(8.7%)	8(17.8%)	0(0)	0.122
Acute ischemia limb	2 (2.8%)	0	0	0	0.373
Respiratory failure	5 (6.9%)	5 (21.7%)	7 (15.6%)	2 (12.5%)	0.237
Heart failure	4 (5.6%)	1 (4.3%)	4 (8.9%)	0	0.441
Ischemic colitis	1 (1.4%)	0	1 (2.2%)	0	0.736
Vocal cord palsy	1 (1.4%)	0	2 (4.4%)	0	0.432
Hepatic failure	1 (1.4%)	1 (4.3%)	1 (2.2%)	0	0.731
Mediastinitis	1 (1.4%)	0	2 (4.4%)	0	0.432
Hospital stay	25.9±33.5	39.0±53.9	33.8±29.7	24.1±17.2	0.323

* p-value for individual Chi-square test.

Overall in-hospital mortality was 22.4% (35/156): 13 patients in Group I, 9 in Group II, 12 in Group III, and 1 in Group IV. Nine patients experienced postoperative cardiogenic shock, 8 experienced hypovolemic shock (uncontrolled bleeding), 6 experienced visceral organ malperfusion, 2 experienced intracranial hemorrhage, 5 experienced cerebral infarction, and 5 experienced sepsis due to pneumonia. Among 121 patients who survived and were discharged, 5 died within the first year of follow-up: 3 because of sepsis due to pneumonia, 1 because of acute myocardial infarction, and 1 because of intracranial hemorrhage. Seven patients could not be followed up because of their distance from the medical center but were confirmed to be alive over a telephonic follow-up. Among the 109 patients followed up postoperatively (14 patients followed up for <1 year), the mean follow-up period was 3.92 ± 2.59 years (median 3.48 years).

The incidence of stroke was higher in Groups II and III; however, the incidences of other complications including acute kidney injury, ischemic bowel, re-exploration for bleeding, and hospital stay did not differ significantly among the 4 groups.

### Factors associated with in-hospital mortality and surgery-related stroke

Table 3 summarizes results of univariate and multivariate regression analysis of factors possibly associated with in-hospital mortality and surgery-related stroke.

**Table 3:** Logistic regression analysis for factors associated with in-hospital mortality (N = 156) and surgery-related stroke (N = 120).

Variables(Number)	n/N(%)	Univariate	P	Multivariate	P
		Crude OR(95%CI)		Adjusted OR(95%CI)	
Mortality (total 35)					
Older age(≧65)	18/47(38.3%)	3.36(1.54–7.35)	0.002	5.78(1.97–16.99)	0.001
Malperfusion					
Cardiac	14/30(46.7%)	4.38(1.86–10.31)	0.001	4.28(1.45–12.66)	0.009
Cerebral	7/26(26.9%)	1.34(0.51–3.51)	0.549	#	
Visceral (Liver/intestine)	8/13(61.5%)	6.87(2.08–22.67)	0.002	14.02(2.95–66.64)	0.001
Renal	3/15(20.0%)	0.85(0.23–3.20)	0.812		
Lower limb	3/13(23.1%)	1.04(0.27–4.01)	0.954	#	
Root reconstruction	10/28(35.7%)	2.29(0.94–5.56)	0.068	3.85(1.22–12.15)	0.021
Total arch replacement	21/68(30.9%)	2.36(1.10–5.09)	0.028	3.31(1.22–8.99)	0.019
Descending aorta stent grafting	13/61(21.0%)	0.90(0.41–1.95)	0.787	**#**	
Surgical groups					
I	13/72(18.1%)	reference		**#**	
II	9/23(39.1%)	2.92(1.04–8.18)	0.042		
III	12/45(26.7%)	1.65(0.68–4.03)	0.271		
IV	1/16(6.3%)	0.30(0.04–2.50)	0.267		
Surgery-related stroke (total 15)					
Older age(≧65)	6/35(17.1%)	1.75(0.57–5.34)	0.328	#	
Root reconstruction	0/21(0%)	-	-	#	
Total arch replacement	12/48(25.0%)	7.67(2.03–28.92)	0.003	7.81(1.77–34.47)	0.007
Descending aorta stent grafting	9/48(18.8%)	2.54(0.84–7.67)	0.099	0.97(0.27–3.50)	0.956
Surgical groups					
I	3/58(5.2%)	reference		#	
II	3/14(21.4%)	5.0(0.89–28.10)	0.068		
III	9/34(26.5%)	6.6(1.65–26.49)	0.008		
IV	0/14(0%)	0(0)	0.999		

# Not included in multivariate model; CI: confidence intervals; OR: odds ratio.

Advanced age (≥65 years), cardiac malperfusion with cardiogenic shock, visceral (liver and intestine) malperfusion, root replacement, and TAR were significantly associated with in-hospital mortality (adjusted OR: 5.78, 4.28, 14.02, 3.85, 3.31 and 95% CI: 1.97–16.99, 1.45–12.66, 2.95–66.64, 1.22–12.15, 1.22–8.99, respectively).

For assessing surgical factors associated with postoperative stroke, we excluded 36 patients with documented preoperative CVA or cerebral malperfusion from the analysis. A total of 15 patients experienced new-onset stroke postoperatively, including 3 patients in Group I, 3 in Group II, and 9 in Group III.

Only TAR was found to be significantly associated with surgery-related stroke (adjusted OR: 7.81, 95% CI: 1.77–34.47, p = 0.007). Descending aorta stent grafting did not increase the risk of surgery-related stroke on univariate and multivariate analyses.

Although some surgical groups were associated with in-hospital mortality (Group II) or surgery-related stroke (Group III) on univariate analysis, the surgical group was not a significant factor when TAR was taken as a confounding factor in multivariate regression analysis.

### Descending aortic remodeling

Data of 95 patients who were followed-up for >1 year were used to evaluate aortic remodeling. Fifty patients had PFL, including 37 patients in Group I, 6 in Group II, 2 in Group III, and 5 in Group IV.

Table 4 summarizes the results of univariate and multivariate regression analyses of factors associated with PFL 1 year after surgery.

**Table 4:** Logistic regression analysis for factors associated with patent false lumen after one year (N = 95).

Variables (Number)	n/N (%)	Univariate		Multivariate	
		Crude OR(95%CI)	p	Adjusted OR(95%CI)	p
Patent false lumen (total 50)					
Tear resection	32/68(47.1%)	0.44(0.18–1.13)	0.088	#	
Root reconstruction	11/16(68.8%)	2.26(0.72–7.09)	0.164	#	
Total arch replacement	8/32(25.0%)	0.17(0.06–0.43)	0.000	0.31(0.10–0.94)	0.038
Descending aorta stent grafting	7/37(18.9%)	0.08(0.03–0.22)	0.000	0.11(0.04–0.32)	0.000
Surgical groups					
I	37/48(77.1%)	reference		#	
II	6/10(60.0%)	0.45(0.11–1.87)	0.269		
III	2/22(9.1%)	0.03(0.01–0.15)	0.000		
IV	5/15(33.3%)	0.15(0.04–0.53)	0.003		

# Not included in multivariate model; CI: confidence intervals; OR: odds ratio

Both TAR and descending aorta stent grafting were significantly associated with a decreased incidence of PFL (adjusted OR: 0.31, 0.11 and 95% CI: 0.10–0.94, 0.04–0.32, respectively). However, tear resection did not reach significance to promote false lumen thrombosis in our analysis (p = 0.088).

**Fig 1:**
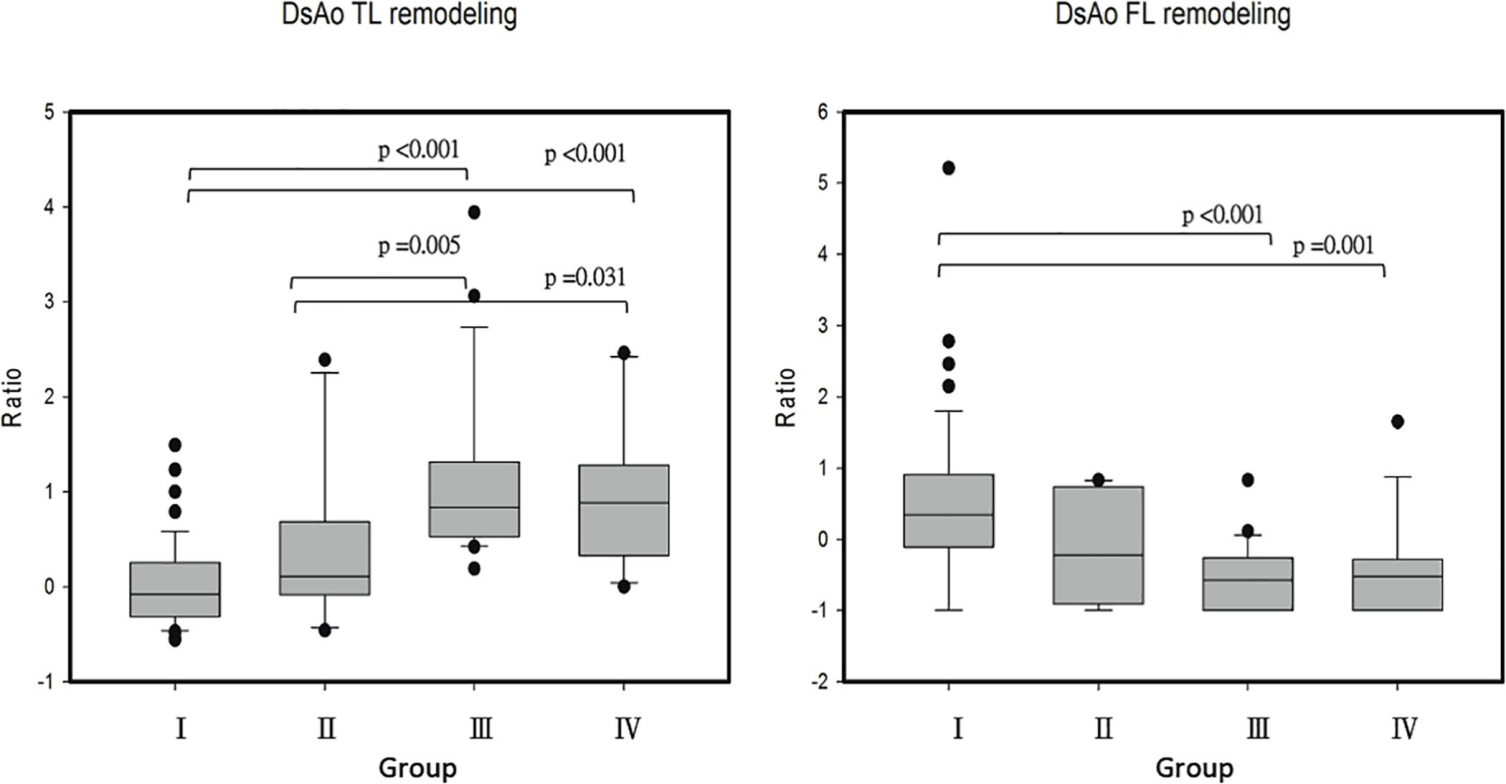
Volumetric change ratio of true (DsAo TL) and false (DsAo FL) lumen of descending aortic segment at one-year postoperative in four surgical groups. Box plots show median and interquartile ranges of remodeling ratios. Group I: proximal aortic replacement (PAR) only; Group II: concomitant total arch replacement (TAR); Group III: concomitant TAR extending with stent graft; Group IV: PAR with descending aorta stent grafting.

Fig 1 shows measures of 1-year volumetric change in true and false lumens of the descending aorta across the 4 groups. The true lumen volume increased by 3% in Group I, 37% in Group II, 113% in Group III, and 94% in Group IV. Both Groups III and IV showed significant true lumen expansion compared to Group I and II. A 48% change in the false lumen was seen in Group I, −10% in Group II, −51% in Group III, and −44% in Group IV. Both Groups III and IV had significant false lumen shrinkage compared to that in Group I.

## Discussion

In this study, in-hospital mortality was 22.4%, with 12.5% new-onset stroke postoperatively. Age ≥65 years, cardiogenic shock, visceral organ malperfusion, root reconstruction, and TAR were significantly associated with in-hospital mortality. TAR was also significantly associated with surgery-related stroke.

### Survival

With regard to survival analysis, results of this study are in line with those of earlier studies that operative mortality for acute type A aortic dissection is affected by age, hemodynamic stability, neurologic status, and presence of malperfusion syndromes [1, 13–15]. Concomitant TAR was also significantly associated with in-hospital mortality in our analysis, and this result is similar to that of earlier reports that PAR in acute DeBakey type I aortic dissection is associated with lower early mortality and complications when compared with aggressive concomitant TAR [16]. At 22.4%, our mortality rate is higher than that in previous studies but comparable to that by the Taiwan National Health Insurance database [17]. The higher mortality is likely a consequence of a complex interaction of multiple factors in Taiwan, including the lack of a specific team managing these urgent patients (only on-duty surgeons), no dedicated operation rooms for these patients, resulting in long wait times, even in other hospitals in Taiwan. Furthermore, physicians are paid for service on a case-by-case basis per the Taiwan National Health Insurance system, which perhaps leads to surgeons operating on patients with poor prognosis. The preoperative malperfusion rate at 41% was higher than that in earlier studies (16%-33%) [1, 18]. This indicates that these patients were more critical, with likely poorer outcomes. The coronary malperfusion and/or infarction rate at 19.2% was also higher than that in the literature (10%-15%) [1]. This corroborates the association between higher hospital mortality and aortic root reconstruction seen in our analysis.

### Neurological outcomes

Advances in surgical techniques, such as the branch-first technique (or arch first) during arch reconstruction [19] or antegrade cerebral perfusion, have shortened cerebral ischemia time and improved neurological outcomes [14, 15]. Applying these techniques in our practice, reconstruction of arch vessels is performed carefully, especially when dissection involves these vessels. However, prolonged cerebral protection time is inevitable. The time of circulatory arrest and cardiopulmonary bypass is also prolonged, which may increase the risk of stroke, paraplegia, and mortality compared to that with PAR only [20, 21]. Our result that TAR significantly increased the risk of surgery-related stroke is in line with these observations. Faulty anastomosis between the supra-aortic arteries and trifurcated vascular prosthesis (anastomosis stenosis, distal PFL, or kinked prosthesis after weaning off of cardiopulmonary bypass) may cause postoperative cerebral malperfusion and increase the risk of surgery-related stroke.

Although the aggressive approaches (Groups II and III) had a positive effect on aortic remodeling, they might have increased risks of mortality and stroke. The method in Group IV maintained the advantage of lower mortality and stroke risk in addition to providing beneficial effects in aortic remodeling. The observation that implanting a stent graft in the dissected aorta may be harmful raises concerns; however, no new re-entry were seen during follow-up. On the contrary, some postoperative patent flow of the false lumen outside the stent graft was seen to have thrombosed during follow-up. The choice of diameter of the stent graft would be an important factor. In our practice, only 5%-10% of the outermost diameter of the proximal descending aorta is added; however, additional experience is necessary. Furthermore, branched thoracic endovascular aortic repair would be possible in the near future and will help mitigate this issue.

### Aortic remodeling

In this study, stent grafting in the descending aorta was also an important factor associated with decreased risk of PFL in addition to TAR. A previous study suggests that the primary tear must be resected whenever possible to depressurize the distal false lumen and decrease the risk of descending aortic aneurysm [22]. Hence, aggressive concomitant TAR is encouraged when the primary tear is located in the arch. However, some reports suggest that PFL does not correlate with successful exclusion of the primary intimal tear but depends on the presence of distal fenestrations between true and false lumens [23]. This implies that, in the initial post-operative period, the true lumen may be compressed by the false lumen where there is a distal large fenestration, leading to complication with PFL if aortic tissue is fragile.

Our results corroborate observations from the computational fluid dynamics simulation model, which clearly reveals significant differences in pressure between true and false lumens, resulting in compression of the true lumen by the false lumen, and false luminal aneurysm in the descending aorta [24]. If the true lumen is expanded by the stent graft, pressure gradients might reduce and blood flow might increase in the true lumen and decrease in the false lumen, resulting in better remodeling of the proximal descending aorta. The risk of PFL (33.3%) in Group IV was lower than that in Group II (60%). Group III which combined 2 significant factors of false lumen thrombosis has the least incidence of PFL (9.1%); however, the risks of mortality and stroke were higher.

Studies have shown that the expansion rate is approximately 1–2 mm per year in patients with PFL when the initial diameter of the proximal descending aorta is <4 cm [25, 26]. Furthermore, approximately 2%-13% of patients require late elective repeat surgery within 5 years, usually with low mortality [1, 25, 26]. Therefore, descending aorta stent grafting in addition to PAR might lower both the expansion and repeat surgery rates because of decreased pressure gradients between true and false lumen and even persistent PFL.

The mortality rate of repeat surgery for aneurysmal enlargement of residual type A aortic dissection was 14.7% (5/34) at our hospital during the same period (unpublished data), which is significantly lower than that in Group II. Hence, in our opinion, concomitant TAR should be reserved for specific situations, such as an enlarged arch during surgery or Marfan syndrome.

In real-world practice, TAR with or without stent graft for acute aortic dissection is associated with significantly higher operative mortality and neurological complications than PAR. Regardless of long-term advantages favoring extensive repair in cases of acute aortic dissection, patients must still survive extensive surgery [27]. Therefore, echoing the opinions of other authors [28], we suggest that TAR should not be considered as the primary approach for all patients and that the approach must be tailored to the surgeon’s and center’s experience and patient’s presentation.

### Study limitations

This was a retrospective, observational study with a small number of patients. The follow-up period was relatively short, and outcomes might not apply to long-term results. Long-term complications (e.g., repeat surgery) could not be addressed, and many factors (e.g., well-controlled hypertension, number of tears, strength of aortic tissue, surgeon’s preference) were not evaluated.

## Conclusion

PAR should be considered in the absence of strong indications for a more extensive repair as it is associated with fewer complications. Aggressive TAR should not be performed routinely. Stent graft implantation in the proximal descending thoracic aorta may benefit aortic remodeling, and it does not increase the risk of mortality and neurological complications when combined with PAR.

## Supporting information

S1 Dataset Type1aorticdissection 156. (SAV)
